# A case report of three synchronous tumors in the same pancreatic specimen

**DOI:** 10.1016/j.amsu.2019.07.001

**Published:** 2019-07-09

**Authors:** A. Giuliani, G. Lazzarin, L. Romano, G. Coletti, V. Vicentini, Walid A. Fatayer M, M. Schietroma, S. Valiyeva, F. Carlei

**Affiliations:** a Department of General Surgery. Department of Biotechnological and Applied Clinical Sciences, University of L'Aquila, L'Aquila, Italy; b UOC Anatomia Patologica, ASL1 Abruzzo, Ospedale San Salvatore, L'aquila, Italy

**Keywords:** Duodenal adenocarcinoma (DA), Pancreatic neuroendocrine tumor(PanNET), Ectopic pancreatic tissue of the duodenum (EPT), Multiple pancreatic tumors

## Abstract

It is known that Duodenal adenocarcinoma (DA) is a rare malignant solid tumor that cause occlusion symptoms with orthodox dysphagia when locally advanced. Pancreatic neuroendocrine tumors (PanNETs) account for about 2% of all pancreatic neoplasms. The combination of these two lesions, with the synchronous presence of ectopic pancreatic tissue (EPT) of the duodenum, has never been described in literature, to our knowledge. Here we report a case of combined DA, EPT and PanNET affecting a 71-year-old woman.

## Highlights

• Simultaneous presence of Duodenal adenocarcinoma pancreatic neuroendocrine tumor ectopic pancreatic tissue of duodenum.
• Biochemical tests did not reveal abnormalities: CA 19-9 was normal, CEA was 153 U/L.
• Ultrasonography confirmed a nodule dislocating pancreas investigated with contrast-enhanced CT and esophagogastroduodenoscopy.
• The association between duodenal adenocarcinomas, PanNET and ectopic pancreatic tissue (EPT) of the duodenum is really rare.

## 1 Introduction

We report a rare case of simultaneous presence of Duodenal adenocarcinoma (DA), pancreatic neuroendocrine tumor (PanNET) and ectopic pancreatic tissue (EPT) of the duodenum [[1], [2], [3], [4], [5], [6], [7], [8], [9], [10], [11]].

This case report is in line with Surgical Case Report (SCARE) guidelines [12].

## 2 Presentation of case

A 71-year-old Caucasian female was admitted to our surgical department for a solid nodule in the second portion of the duodenum in November 2018. Past medical records revealed several dyspepsia and epigastric nocturnal pain. She had history of type 2 diabetes treated with metformin, arterial hypertension treated with betablockers, no smoking or alcohol abuse and did not use any drug on regular basis. There was no family history of duodenal or pancreatic neoplasms. Biochemical tests did not reveal abnormalities: CA 19-9 was normal (<37 U/mL), but CEA was >100 ng/mL (153 U/L).

**Fig. 1:**
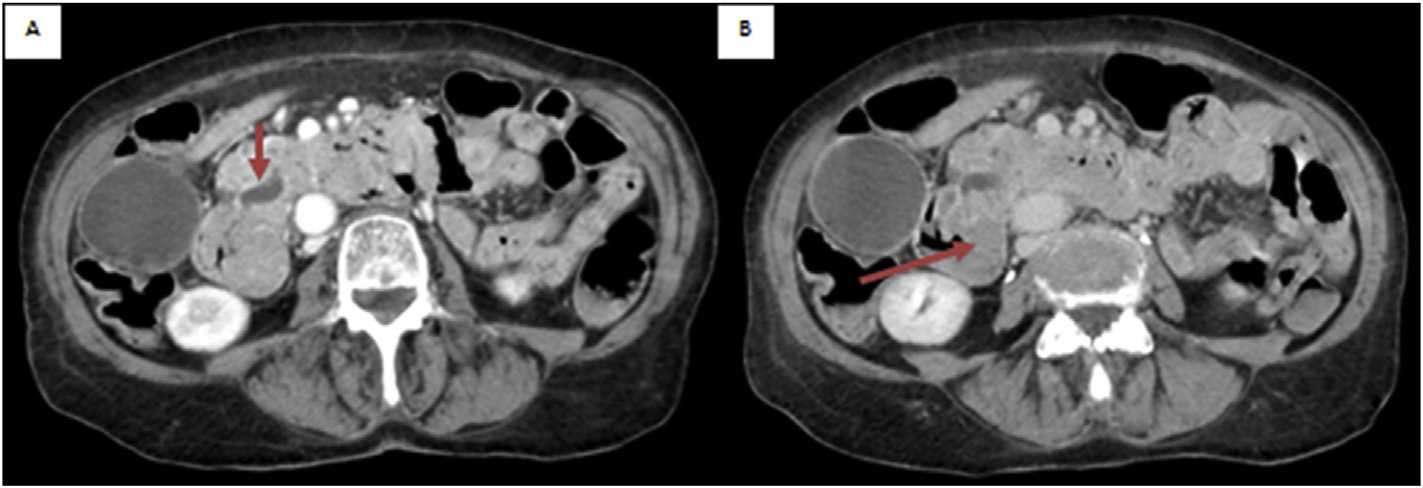
Duodenal adenocarcinoma shown on axial CT-scan during the portal phase (A and B). CT images show the marked thickening of the medial wall of the second part of the duodenum (B, arrow). The lesion had a central indentation and raised, rolled edges. In figure A (A, arrow) is evident the obstruction of the Vater papilla with dilatation of Wirsung duct.

An Ultrasonography confirmed the presence of a solid nodule dislocating the pancreas, which was further investigated with contrast-enhanced CT-scan (CT) (Fig. 1A and 1B) and esophagogastroduodenoscopy. All the instrumental findings supported the diagnosis of solid tumor of the second portion of the duodenum conditioning orthodox dysphagia and occlusion symptoms.

Due to the symptoms and the suspected diagnosis of duodenal neoplasia, the patient then underwent Pancreaticoduodenectomy (DCP). We performed a classic Whipple procedure: the duodenum and head of the pancreas were separated from the retroperitoneal bed medially past the aorta and distally to the ligament of Treitz. The gallbladder was removed. The bile duct was dissected free from the adjacent portal structures and divided above the cystic duct entry across the common hepatic duct. The gastroduodenal artery was twice ligated to minimize the chance of subsequent erosion and bleeding. Lymph nodes anterior to the proper hepatic artery were taken with the specimen. After that, left gastric and gastroepiploic vessels were divided at the gastric wall and the stomach was divided across the proximal antrum with a stapler. Transection of the pancreas in front of the portal vein was the next surgical step. The transverse colon and its mesentery were elevated cephalad, and the entire small bowel was eviscerated to facilitate the dissection of the distal duodenum, proximal to the ligament of Treitz. The jejunum was divided with a stapler 10 cm past the ligament of Treitz. The end of the jejunum was oversewn with nonabsorbable sutures over the staple line and it was brought through the right side of the transverse mesocolon. The transmesocolic pancreatico-jejunostomy was performed first, using an outer row of interrupted nonabsorbable sutures that included most of the cut surface of the pancreas and an inner row of interrupted synthetic absorbable sutures duct to mucosa. The anastomosis was completed with an anterior row of silk sutures. Distal to the pancreatic anastomosis, an end-to-side hepatico-jejunostomy was made with a single layer of interrupted closely spaced synthetic absorbable sutures. After fixing the jejunal loop to the transverse mesocolon with interrupted nonabsorbable sutures, gastrointestinal continuity was restored with an antecolic Hofmeister-type Billroth II gastro-jejunostomy. This anastomosis was made with running absorbable sutures as an inner layer and interrupted nonabsorbable sutures as an outer layer.

The nasogastric tube was removed on postoperative day 1, and clear liquids was allowed on day 2. The diet was advanced to low-fat soft solids. Blood glucose was monitored from day 1, such as the concentration of amylase in the drainage (measured on day 1 and 5).

Post-operative course was featured by grade A pancreatic fistula [biochemical leak - according to ISGPS 2016 guideline [13]], without fever, abdominal pain or leukocytosis. Two surgical drainages was removed on the 5th postoperative day. The patient was discharged on the 7th postoperative day.

We performed a follow-up visit on 14th postoperative day, that confirmed good general health conditions, without surgical complications. Than the patient underwent to adjuvant first line chemotherapy and was followed by oncological department of our hospital.

## 3 Histopathologic analysis

**Fig. 2:**
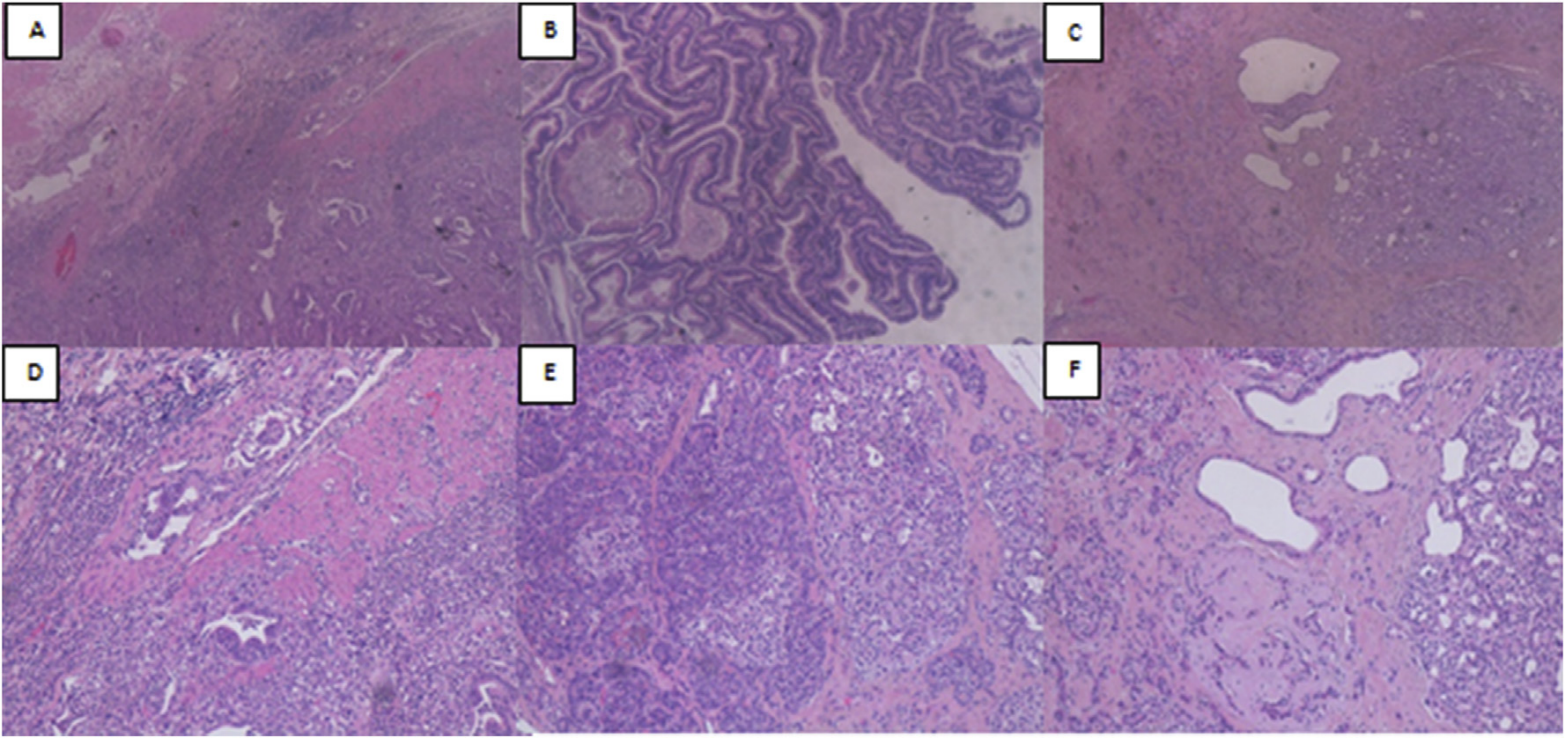
The Duodenal adenocarcinoma infiltrate the muscularis propria (A: H&E stain, 4X OM; D: H&E stain, 10X OM); the tubulo-villosus adenoma of the duodenum (B: H&E stain, 4X OM); the pancreatic neuroendocrine tumor NET, G1 (C: H&E stain, 4 OM; F: H&E stain, 10X OM) was characterized by a solid-trabecular histologic pattern; the ectopic pancreatic tissue on the serosa of the duodenum, with nodular pancreatic neuroendocrine microadenomatosis (E: H&E stain, 10X OM).

Pathological macroscopic examination revealed that Vater's papilla was entirely occupied by an exophytic mass of 11 cm of major diameter; microscopic examination confirmed the diagnosis of moderately differentiated duodenal adenocarcinoma (Fig. 2 A and D) on tubulo-villous adenoma (Fig. 2 B), infiltrating the duodenal wall and focally, the periduodenal adipose tissue; peritumoral lymphovascular invasion was present; metastasis in 3 of 26 duodenal-peripancreatic lymph nodes were detected (pT2pN1 sec. AJCC 8th).

Two other neoplasms were discovered: at about 9 cm of distance from exophytic mass, we discovered a whitish nodular formation measuring about 2 cm of greater diameter, on the serosa of duodenum. Microscopic examination described it as a nodule of ectopic pancreas with nodular pancreatic neuroendocrine microadenomatosis (Fig. 2 E).

Finally, in the head of the pancreas, a well differentiated pancreatic neuroendocrine tumor (Fig. 2C and 2 F) 2.6 cm of larger diameter, was found; Ki-67 was positive in less than 3% of neoplastic cells, mitotic count < 2/10 high powered fields (HPF). It was characterized by stromal invasion and lymphovascular aspects with diffuse positivity of synaptophisin (pT2pN0 sec. AJCC 8th).

In gastric and ileal surgical resections no neoplastic tissue was found.

## 4 Immunohistochemistry

**Fig. 3:**
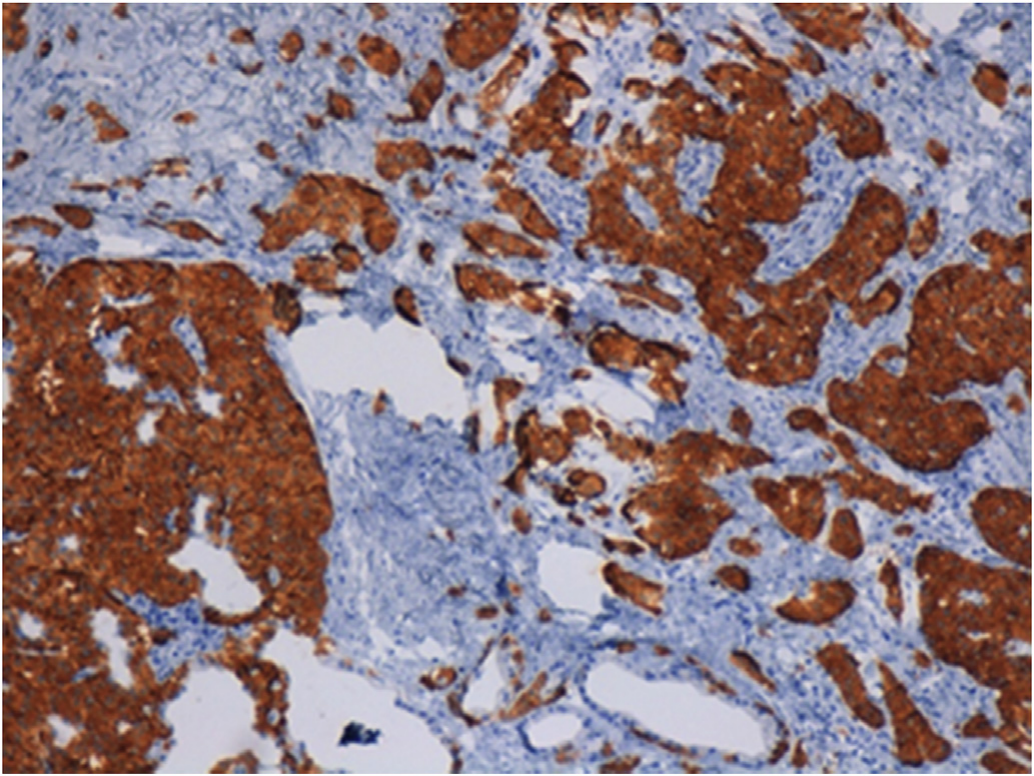
Immunohistochemistry showing high positivity for synaptophysin of well differentiated pancreatic neuroendocrine tumor (Synaptophysin IHC, 10X OM).

Immunohistochemical analysis was performed as previously described [14,15]. Briefly, using 4 μm formalin-fixed paraffin-embedded sections, immunohistochemical analysis was conducted with the standard polymer system and peroxidase methods. After heat-induced, antigen retrieval with a heated plate and 0.01 mol/L of citrate buffer, pH 8.9, for 15 min, all samples were processed using a sensitive ‘Bond Polymer Refine’ detection system in an automated Bond immunohistochemistry instrument (Vision-Biosystem, Leica, Milan, Italy). Sections incubated without the primary antibody served as negative controls. Immunostaining confirmed the diagnosis of pancreatic neuroendocrine tumor (Fig. 3) with focal positivity for chromogranin A and high positivity for synaptophysin, as well as for CD56 (Thermo Scientific, clone:123C3.D5, 1:100 dilution), with a Ki67 proliferative index 3% (Novocastra, clone:MM1, 1:50 dilution).

## 5 Discussion

To our knowledge, this is a rare report on the simultaneous presence of three different neoplasms in the same pancreatic specimen in a patient.

Duodenal adenocarcinomas (DA) are rare tumors accounting for 0.5% of all gastrointestinal tumors and for 10% of all periampullary neoplasms [16]. Indeed, depending on the insurgence site, DAs are classified like periampullary duodenal adenocarcinomas and extra-ampullary adenocarcinomas (third or fourth portion of the duodenum). It seems that periampullary adenocarcinomas are most frequent, but literature reports are insufficient. This lesion can directly originate from duodenal mucosal epithelium, or can originate from preexistent benign lesion, such as duodenal adenoma (as described in our case), familial adenomatous polyposis (FAP), lymphoid iperplasia, Peutz-Jeghers syndrome. Since patients do not typically came to visit until tumors have grown to sufficient size to cause symptoms, the diagnosis of DA is difficult and often delayed. When symptoms do appear, they are nonspecific and include: abdominal pain (the most common symptom - 17), nausea, vomiting, fatigue, weakness, and weight loss. Anemia, gastrointestinal obstruction and jaundice are symptoms associated with advanced disease [17,18]. Endoscopy is the preferred diagnostic modality as it allows simultaneous visualization and biopsy [17]. Contrast-enhanced computed tomography is important for assessing involvement of nearby structures, determining resectability and planning surgery [19]. Tumors located in the second portion of the duodenum typically require pancreaticoduodenectomy (PD) because of proximity to head of the pancreas, distal bile duct and ampulla of Vater [20]. The importance of an adequate lymphadenectomy cannot be underscored to improve stage stratification and prognostication. Unfortunately, surgery for DA can be associated with significant morbidity and mortality. DA represents an aggressive cancer but in patients with resectable disease, long term outcomes are better than with other periampullary malignancies [21].

PanNETs prevalence as sporadic lesions ranges from 1% to 10% in adult population at autoptic findings [22]. PanNETs measuring less than 0.5 cm are defined microadenomas and they are considered biologically benign. By definition, these early stage PanNETs are nonfunctional [23]. The diagnosis of endocrine microadenomas usually follows the pathological analysis of a pancreatic specimen resected for other reasons. PanNETs are classified as functioning or non-functioning depending on whether they cause hormonal hypersecretion syndrome [24]. Functioning PanNETs result in hormonal hypersecretion syndromes. Non-functioning PanNETs cause nonspecific symptoms, such as vague abdominal pain, and can be an incidental finding. The distinction between functioning and non-functioning PanNETs is based on clinical presentation, and there is no absolute difference in hormone expression between functioning and non-functioning PanNETs [25].

## 6 Conclusion

In conclusion, the association between duodenal adenocarcinomas, PanNET and ectopic pancreatic tissue (EPT) of the duodenum is really rare. This case report seems to be unique, since three distinct neoplastic lesions, have been found in the same patient. Considering the low prevalence of each of these neoplasms, the probability to find them simultaneously is exceedingly rare.

## Ethical approval

No.

## Sources of funding for your research

No funding

## Author contribution

Writers: Lazzarin G, Romano L.

Data collection: Valiyeva S, Vicentini V, Giuliani A.

Images: Coletti G, M Walid A Fatayer, Final Revision: Schietroma M, Carlei F.

## Conflicts of interest

The authors have no conflicts of interest to declare.

## Registration of research studies

1. Name of the registry: no.

2. Unique Identifying number or registration ID: no.

3. Hyperlink to the registration (must be publicly accessible): no.

## Guarantor

Carlei F.

## Consent of patient

Written informed consent was obtained from the patient for publication of this case report and any accompanying images.

## Provenance and peer review

Not commissioned, externally peer reviewed.
